# The Advertisement of Quack Remedies

**Published:** 1907-12-28

**Authors:** 


### The Hospital

A Journal of

The Medical Sciences and Hospital Administration.

New Series. No. 41, Vol. II. [No. 1113, Vol. XLIII.] SATURDAY, DECEMBER 28, 1907.

### The Advertisement of Quack Remedies.

The development of the art and practice of public advertisement is a conspicuous feature of modern commercial activity, and as an almost essential business method it receives a practically universal recognition. Colleges, clubs, and other organisations exist to cultivate it, and on every hand may be seen indications that these efforts are wanting neither in vigour nor in skill. How far these various institutions are concerned with the ethical aspects of the subject we do not know, but, to judge from many conspicuous instances, it may be doubted whether there has yet developed any sustained and serious effort to compel the modern advertiser to accommodate his protestations to the ordinary demands of the moral code. Accepting, however, the practice as a legitimate, and, indeed, a necessary business method, it may be asked with whom lies the responsibility for its occasional abuse and degradation. The immediate responsibility manifestly rests upon the individual advertiser himself, and in some circumstances is confined to him. But a further question arises when publicity is gained through the columns of a newspaper or public journal. For here, at least in some measure, the presentation of the advertisement is a joint undertaking, and one in which those who effect publication take a recognised part. The accurate adjustment of the respective shares of the parties concerned may possibly be a difficult and delicate process. But it will hardly be contested that there are advertisements which no reputable journal would admit to its columns, and this necessarily allows the claim that the publisher cannot altogether escape accepting some responsibility for what he consents to introduce to public attention.

The application of the general principle just defined, it must be admitted, is not altogether a simple and easy task. Probably, indeed, it would be impossible to frame a general rule to which every individual instance could be readily accommodated. Thus, in practical life, it would be absurd to demand that a newspaper manager should guarantee the precise accuracy of the terms of all the advertisements which he publishes. Public opinion sanctions, or at least does not definitely condemn, a certain measure of picturesque exaggeration on the part of those who, having something to sell, aim at obtaining the ear of the public. This is generally both recognised and understood, and it cannot be claimed that a newspaper proprietor ought to insist upon a standard conspicuously in advance of the general opinion of the community. For such a censorship he has neither the time nor the ability, and an attempt to exercise a function of this order would produce serious results not only on his own financial position, but also on public convenience. It therefore follows that while a newspaper cannot escape some responsibility for the advertisements which it publishes, that responsibility is a limited one, and the question in any particular instance is whether what may be called the natural and reasonable limit has been passed.

Now the question here discussed becomes of manifest importance when it is considered in regard to the advertisement of medicines and other proclaimed cures in the columns of the lay press. So far as our own view is concerned, it will be manifest, from what we have already said, that we have no desire to quarrel with any of our contemporaries merely because, through their agency, the public are introduced to many grotesque therapeutic claims. Doubtless, as a result, numbers of people are deceived, and sometimes positive harm is done; but the statement that somebody's pills "touch" some particular organ in the human body is beyond the purview of the newspaper proprietor, and we think it would be unreasonable to ask him to refuse to publish it until a scientific demonstration of its accuracy has been afforded. The same comment applies to many other claims. Ridiculous as numbers of these are to anyone provided with a knowledge of physiology and therapeutics, they are not to the lay mind gross and palpable frauds. But a different position emerges when an advertisement can no longer be covered even by so lame and halting an apology as that just mentioned, and when it advances claims that are openly and notoriously unfounded and inaccurate. Still more serious is the situation if such claims involve the health, and it may be the lives, of individuals who may happen to give heed to them. Here, at all events, a journal must be held culpable, seeing that there are ready methods of obtaining the necessary enlightenment, and that the subject matter of such advertisements is to any educated and well-informed individual conspicuously and unquestionably fraudulent.

We quite admit that to draw the line with precision is not always easy; but there are instances not a few for which even the most generous interpretation can find no excuse. Take, for example, some of the so-called electrical appliances for which such outrageous pretensions are advanced, and which have again and again been proved to be absolute swindles. Advertisements of these are to be met with in journals claiming to be respectable and, some of them at least, boasting an odour of sanctity. And within the last week or so, some of the leading organs of the daily press have welcomed an advertiser who denounces "medical butchery," and claims to cure "every kind of internal inflammation" by "scientific magnetic force from a gifted healer." To publish such pestilent nonsense is to incur some share in the danger it may work, and the journals which receive it ought not to escape public censure and condemnation.

